# Increasing population size can inhibit cumulative cultural evolution

**DOI:** 10.1073/pnas.1811413116

**Published:** 2019-03-14

**Authors:** Nicolas Fay, Naomi De Kleine, Bradley Walker, Christine A. Caldwell

**Affiliations:** ^a^School of Psychological Science, University of Western Australia, Crawley, WA 6009, Australia;; ^b^Behaviour and Evolution Research Group, Psychology, School of Natural Sciences, University of Stirling, FK9 4LA Stirling, United Kingdom

**Keywords:** population size, demography, cumulative cultural evolution, success bias, cultural evolution

## Abstract

The extent to which larger populations enhance cumulative cultural evolution (CCE) is contentious. We report a large-scale experiment (*n* = 543) that investigates the CCE of technology (paper planes and their flight distances) using a transmission-chain design. Population size was manipulated such that participants could learn from the paper planes constructed by one, two, or four models from the prior generation. These social-learning conditions were compared with an asocial individual-learning condition in which individual participants made repeated attempts at constructing a paper plane, without having access to any planes produced by other participants. Larger populations generated greater variation in plane performance and gave participants access to better-adapted planes, but this did not enhance CCE. In fact, there was an inverse relationship between population size and CCE: plane flight distance did not improve over the experimental generations in the 2-Model and 4-Model conditions, but did improve over generations in the 1-Model social-learning condition. The incremental improvement in plane flight distance in the 1-Model social-learning condition was comparable to that in the Individual Learning condition, highlighting the importance of trial-and-error learning to artifact innovation and adaptation. An exploratory analysis indicated that the greater variation participants had access to in the larger populations may have overwhelmed their working memory and weakened their ability to selectively copy the best-adapted plane(s). We conclude that larger populations do not enhance artifact performance via CCE, and that it may be only under certain specific conditions that larger population sizes enhance CCE.

## Significance

Cumulative cultural evolution (CCE)—the social-learning process through which adaptive modifications accumulate over historical time—is crucial to the advancement of the human species, and yet little is known about the factors important to CCE. Larger populations may enhance CCE, although this is contentious. We report a large-scale experiment that manipulates population size and tests its effect on artefact performance using a paper plane construction task. Over the experimental generations, smaller populations showed the strongest improvements in plane performance (indexed by flight distance). We conclude that larger populations do not enhance artefact performance via CCE and that it may be only under certain specific conditions that larger population sizes enhance CCE.

Cumulative cultural evolution (CCE) is the process through which adaptive modifications accumulate over historical time, driving the incremental improvement of material and symbolic artifacts (1–4). Demography may be crucial to CCE: in particular, larger populations provide access to better-adapted artifacts, greater artifact variation, and the opportunity this affords for recombination (5). (By which, we mean members of an interacting “effective” population size, as opposed to a “census” population size in which many of its members are noninteracting.) Henrich (6) suggests that a decline in the complexity of Tasmanian technology following its isolation from mainland Australia (due to rising sea levels between 12–10 kya) can be explained by the consequent reduction in the population of interacting social learners. However, this explanation is contentious. Vaesen et al. (7) cast doubt on the assumptions behind the mathematical models upon which this explanation is based (ref. 6; see also ref. 8) and the archeological and ethnographic record it tries to explain (see also refs. 9 and 10).

Laboratory experiments that manipulate population size and test its causal effect on cultural accumulation provide some support for the benefits of larger populations (11–13). For example, having access to more demonstrators helped maintain cultural complexity [image-editing and knot-tying skill (13)], and being a member of a larger group improved artifact performance [on a virtual fishing net and arrowhead production task (11)]. However, other studies find no evidence that a larger population size enhances CCE. For example, the complexity of folk tales was unaffected by population size (14), and the CCE of technology (the flight distance of paper planes) was unaffected by the number of artifacts to which participants had access (15). So, the experimental evidence supporting a direct link between population size and CCE is equivocal.

These conflicting experimental results may be explained by the greater opportunity for success-biased copying—the preferential copying of successful models—in some experiments compared with others (see ref. 16 for a discussion of the various social-learning strategies). In the study by Derex et al. (11), participants were permitted to view a single artifact produced by a single demonstrator (ranked by their performance score). In the study by Muthukrishna et al. (13), participants were informed of the score associated with each artifact and could view the artifacts produced by any or all of five demonstrators. Participants tended to copy the artifact produced by the most successful demonstrator (Expt. 1) and overwhelmingly chose to view a single artifact produced by a single demonstrator (Expt. 2) (13). So, in both of these studies, participants could select the single most successful demonstrators to learn from and could avoid encountering the artifacts produced by less successful demonstrators. Performance improvements in these experiments may be accounted for by a combination of population size and success-biased copying that was facilitated by the opportunity to filter out less successful variants. So, these experiments do not test the basic effect of population size on CCE.

Success-biased copying may have been more challenging in the study by Caldwell and Millen (15). Here, participants observed each of the artifacts produced by each of the other members of their group. Participants could therefore not avoid encountering the artifacts produced by less successful demonstrators via selective filtering. Human working memory—used to hold information for temporary processing—has a limited capacity of around four chunks of information (17, 18). In the experiment by Caldwell and Millen (15), the artifact (paper plane), its performance (flight distance), and its construction method correspond to at least three chunks of information. It follows that as population size was increased (from one to two to three models), participants’ working-memory capacity may have been overwhelmed, reducing their ability to engage in success-biased copying. In addition, in this experiment, participants were given the same fixed amount of time to study the paper planes produced by the members of their group. This may have disadvantaged participants allocated to the larger populations, as the mean amount of time available to view each plane decreased as the population size increased.

We present a large-scale experiment (*N* = 543) that tests the effect of population size on the CCE of technology. We used the paradigm developed by Caldwell and Millen (1, 15, 19) because it returns a precise measure of artifact performance (paper plane flight distance), but we adapted the procedure to ensure that the mean viewing time per artifact was the same across the different population sizes. In each population size, participants viewed each of the paper planes produced by each member of the prior experimental generation. So, participants could not avoid encountering less successful planes (via filtering), which ensured a “pure” test of population size. This is crucial to establishing the basic effect of population size on the improvement of artifact performance via CCE. An asocial, individual-learning condition was also included. This condition, often missing from experimental studies of CCE (20), provided a baseline against which the social-learning conditions could be compared. First, we tested if larger populations generated greater artifact variation (Hypothesis 1) and gave participants access to better-adapted artifacts (Hypothesis 2), crucial ingredients if larger populations are to enhance CCE. We then tested if larger populations enhanced the improvement in artifact performance via CCE (Hypothesis 3). Finally, we report an exploratory test of the relationship between population size and success-biased copying.

## Results

All of the experimental variables were normally distributed (skewness within ±1.10 and kurtosis within ±1.60) (21, 22). The data were analyzed using linear mixed effects modeling, including by-chain random intercepts. The fixed effects (Condition, Generation) were centered before analysis. (The Individual Learning, 1-Model, 2-Model, and 4-Model conditions were numerically coded: 0, 1, 2, and 4, respectively, the numeric value corresponding to the number of other models from which the participant could socially learn.) All analyses were performed and all figures were created in R (23). Statistical models were estimated using the lmer() function of lme4 (24). The data and R Script are available on the Open Science Framework: https://osf.io/486wy.

We first test that the prerequisites were met for larger populations to enhance artifact performance via CCE, i.e., access to greater artifact variation and better-adapted artifacts. Next, we test if larger populations more strongly improved artifact performance via CCE. Finally, we report an exploratory test of the relationship between population size and success-biased copying. Specifically, we test the extent to which the opportunity for success-biased copying in larger populations was counteracted by constraints on human working memory.

### Prerequisites for Larger Populations to Enhance CCE.

Larger populations can enhance CCE if they provide access to greater artifact variation (Hypothesis 1) and better-adapted artifacts (Hypothesis 2). Artifact variation was operationalized as the range in paper plane flight distances in the 2- and 4-Model conditions (maximum recorded flight distance minus minimum recorded flight distance) at each generation. Because participants in the Individual Learning and 1-Model conditions were exposed to a single plane, there was no variation in plane performance. Artifact quality was operationalized as the maximum flight distance of the planes participants constructed at Generation 1 in each condition. For the Individual Learning and 1-Model conditions, this was the plane built by each participant at Generation 1. For the 2- and 4-Model conditions, this was the furthest-flying of the two or four planes produced by participants at Generation 1. Examining plane flight distance at Generation 1 ensured the artifacts were unaffected by the social-learning condition.

**Fig. 1.**
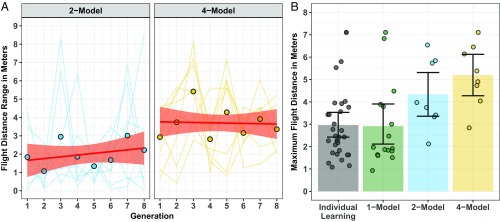
(*A*) The variation in paper plane flight distances (maximum − minimum) across the experimental generations in the 2- and 4-Model conditions (plotted for each chain). The dot points indicate the overall mean at each generation. The red straight line is the linear model fit, and the light red shaded area is the bootstrapped 95% CI. (*B*) The maximum plane flight distance at Generation 1 in the Individual Learning, 1-, 2-, and 4-Model conditions. The colored bars indicate the overall mean for each condition, and the dot points indicate the mean for each chain. Error bars are the bootstrapped 95% CIs.

As predicted, variation in plane performance was higher in the 4-Model condition compared with the 2-Model condition (β *=* 0.85, SE = 0.18, t=4.77,P<0.001). There was no evidence of a statistical change in the variation in plane performance over the experimental generations (P=0.582) or of a condition by generation interaction (P=0.428) (Fig. 1*A*). Also as predicted, the maximum plane flight distance increased as population size increased (β *=* 0.58, SE = 0.16, t=3.71,P<0.001) (Fig. 1*B*). The prerequisites for larger populations to enhance CCE were met: larger populations gave participants access to greater artifact variation, supporting Hypothesis 1, and better-adapted artifacts (at Generation 1), supporting Hypothesis 2. Note that artifact variation and artifact quality (2-Model and 4-Model conditions at Generation 1) were positively correlated, r(16)=0.53,P<0.001, but not strongly enough to be considered collinear.

### Do Larger Populations Enhance Artifact Performance via CCE?

**Fig. 2.**
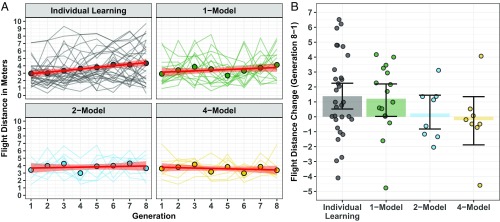
(*A*) The change in paper plane flight distances across the experimental generations in the different conditions (plotted for each chain). The dot points indicate the overall mean at each generation. The red straight line is the linear model fit, and the light red shaded area is the bootstrapped 95% CI. (*B*) The change in plane flight distance across the experimental generations (Generation 8 − Generation 1). The colored bars indicate the overall mean for each condition, and the dot points indicate the mean for each chain. Error bars are the bootstrapped 95% CIs.

Twenty-five outliers (3.29% of data) were identified using the Interquartile Range rule (25). To estimate effects that would be more replicable, the outlying observations were Winsorized to the 95th percentile, done at each level of generation (26). Note that the same pattern of results was returned by the unadjusted flight-distance scores. The best-fitting model specified Condition and Generation as fixed effects with interaction (β *= −*0.06, SE = 0.02, t=−3.66,P<0.001) (Fig. 2*A*). Comparison of the Individual Learning and 1-Model conditions indicated an increase in plane flight distance over the experimental generations (by 1.38 and 1.21 m from Generation 1–8, respectively; β *=* 0.17, SE = 0.04, t=4.79,P<0.001; Fig. 2*B*). By contrast, there was no statistical evidence of an increase in plane flight distance in the 2- and 4-Model conditions (0.22 and *−*0.23 m from Generation 1–8, respectively; β *= −*0.02, SE = 0.05, t=−0.46,P=0.649; Fig. 2*B*). Larger populations gave participants access to greater variation in paper plane performance and better-adapted planes (at Generation 1) but did not enhance artifact performance via CCE. We therefore reject Hypothesis 3.

### Why Did Artifact Performance Not Improve via CCE in the Larger Populations?

Success-biased copying predicts that the degree to which a variant is copied will be associated with its quality. However, if working memory is overwhelmed by the greater number of variants participants have access to in larger populations, this will lower transmission fidelity and weaken success-biased copying. This hypothesis was tested using plane flight distance (at Generation N) to predict plane similarity (between Generation N and Generation N + 1).

Photographs of each paper plane were taken from the same angle at each generation. Pairs of photographs at Generation N and Generation N + 1 were presented on a computer screen and were rated (by author NDK) for similarity on a 10 point Likert-type scale, from 0 (“extremely dissimilar”) to 9 (“practically identical”). In total, 1,449 pairs of photographs were rated for similarity. A subset (678 pairs of planes; randomly sampled from each condition) were rated for similarity by a second judge. The raters were blind to the condition and generation from which each plane was sampled. Comparison of the two sets of plane similarity ratings showed acceptable intercoder agreement [r(677)=0.59,P<0.001].

**Fig. 3.**
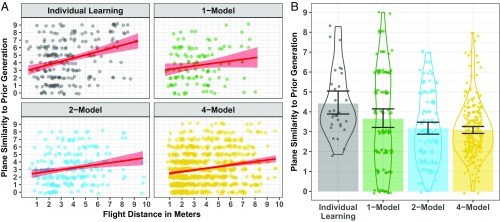
(*A*) The relationship between paper plane flight distance (in meters, at Generation N) and plane similarity (Generation N and Generation N + 1) in the different conditions (plotted for each participant). The red straight line is the linear model fit and the light red shaded area is the bootstrapped 95% CI. (*B*) The plane similarity ratings in the different conditions. The colored bars indicate the overall mean for each condition, and the dot points indicate the mean for each participant. Error bars are the bootstrapped 95% CIs.

The plane similarity ratings were entered into a linear mixed-effects model with Condition and Generation N plane flight distance as fixed effects. The best-fitting model specified Condition (β *= −*0.32, SE = 0.06, t=−4.09,P<0.001) and Generation N plane flight distance (β *=* 0.25, SE = 0.03, t=8.24,P<0.001) as fixed effects without interaction (Fig. 3*A*). Consistent with success-biased copying, plane flight distance (at Generation N) predicted plane similarity (between Generation N and Generation N + 1). The absence of an interaction effect (P=0.189) indicates that the influence of success-biased copying was comparable across the experimental conditions. This shows that, contrary to a random drift account, in all conditions participants’ copying behavior was strategic. Increased population size lowered transmission fidelity; as population size increased plane similarity between the adjacent generations decreased (Fig. 3*B*). This supports our exploratory hypothesis that working-memory constraints weakened copying fidelity as population size was increased.

## Discussion

The positive effect of larger populations on CCE is contentious (7, 9, 10). Using a large-scale experiment (*n* = 543), in which participants were unable to filter out less successful artifacts, we investigated the pure effect of population size on the CCE of technology (paper planes). As predicted, larger populations generated greater artifact variation—conferring more opportunity for artifact recombination—and provided access to better-adapted artifacts. However, this did not enhance CCE. In fact, there was an inverse relationship between population size and CCE, such that an improvement in artifact performance was not observed in the 2- or 4-Model social-learning conditions but was observed in the 1-Model social-learning condition. Performance improvements in the 1-Model social-learning condition were comparable to those in the Individual Learning condition. We then tested the speculative hypothesis that working-memory constraints may have reduced copying fidelity, weakening success-biased copying in the larger populations. This hypothesis was supported; success-biased copying was evident in all conditions, but overall copying fidelity decreased as population size increased. In addition, plane complexity (indexed by the number of folds) increased as population size increased (*SI Appendix*). Artifact recombination and an associated increase in artifact complexity may also have contributed to the lower copying fidelity in the multiple models conditions (27). Further research is needed to tease apart these explanations.

The finding that increasing population size did not enhance artifact performance via CCE is consistent with research on group performance and decision-making (28). As group size increases, the coordination problem faced by its members also increases, thereby reducing group productivity relative to its potential (29–31). For example, in brainstorming groups, the mean number of ideas generated per person decreases as group size is increased (32). There is no such coordination problem in the Individual Learning condition, which may explain why participants in this condition performed as well as those in the 1-Model social-learning condition and better than those in the 2- and 4-Model social-learning conditions. As suggested by Miton and Charbonneau (20), the Individual Learning condition and the social-learning conditions were matched in terms of total learning time. However, the conditions were mismatched in terms of the participants’ task experience; participants in the Individual learning condition constructed a paper plane eight times, whereas participants in each of the social-learning conditions constructed a paper plane once over the course of the experiment. We therefore recommend caution about direct comparisons between the Individual Learning condition and the social-learning conditions. Our results nevertheless emphasize the importance of individual trial-and-error learning to artifact innovation and adaptation (33).

Our findings do not rule out the importance of larger populations to CCE. However, in the context of the experimental paradigm used—a transmission-chain design that included relatively brief and sequential inspection of the artifacts produced by members of the prior generation—our findings indicate that a larger population size alone may not be sufficient to enhance artifact performance via CCE. In fact, our results suggest that larger populations may inhibit CCE due to the working-memory constraints and coordination problems inherent to larger groups. We propose that it may be only under certain specific conditions that larger population sizes enhance CCE. Network structure, or connectivity, may be crucial (34–37). Although people regularly interact in groups, the basic arena for social interaction is the dyad (38). A major advantage of dyadic interaction is that it minimizes the aforementioned working-memory and coordination constraints, allowing participants to dedicate their cognitive resources to evaluating the information presented. Dyadic interaction may be the dominant context for social interaction because it is optimally adapted for information exchange.

Language-evolution experiments indicate that larger population sizes enhance CCE when member interactions are organized in dyads (37, 39, 40). The communication systems that evolved in larger populations (organized as a series of separate dyadic interactions) were optimized for effective and efficient communication and were easier to acquire and were transmitted with higher fidelity by subsequent generations compared with the communication systems that evolved in smaller populations. In these experiments, because participants interacted in dyads, they had the cognitive resources available to assess the informational value of the single communication variant produced by their partner and adopt it or discard it based on this evaluation. By contrast, in the present experiment, members of the large 4-Model populations had to assess four variants before (potentially) adopting any, which may have overwhelmed their cognitive resources. In the language-evolution experiments, when dyadic interactions were iterated across a large population of agents, a copy-if-better strategy (41–43) led to the survival and propagation of communication variants that were well adapted for both use and acquisition (44). A similar benefit of larger populations is observed in a naturalistic study of linguistic systems (45).

## Conclusion

A recent review of the archeological record argues against a reduction in cultural complexity in Tasmania following its isolation from mainland Australia (46). It points out that although simple bone tools were lost, other, more complex technologies, such as stone tools, clothes, and canoes, were then manufactured in Tasmania. In addition, an analysis of other hunter-gatherer societies did not support the population size hypothesis. These ethnographic and archeological data align with the experimental data reported here.

In the experiment reported, larger populations generated greater artifact variation and gave participants access to better-adapted artifacts, but this did not lead to an improvement in artifact performance via CCE. In fact, there was an inverse relationship between population size and CCE: there was no improvement in paper plane flight distance over the experimental generations in the 2-Model and 4-Model social-learning conditions, but there was an improvement in the 1-Model social-learning condition. The improvement in plane flight distance in the 1-Model social-learning condition was comparable to that in the asocial Individual Learning condition, highlighting the importance of individual trial-and-error learning to artifact innovation and adaptation. An exploratory analysis indicated that having access to more artifacts may have overwhelmed participants’ working memory in the larger populations, reducing their ability to selectively copy the more successful artifacts. In the context of the present experiment, artifact performance improved via CCE in the small populations but not in the large populations, indicating that other factors may be important for large populations to enhance CCE.

## Methods

The study received approval from the University of Western Australia Ethics Committee. Participants viewed an information sheet before giving written consent to take part in the study. The information sheet and consent form were both approved by the Ethics Committee. All methods were performed in accordance with the guidelines from the National Health and Medical Research Council/Australian Research Council/University Australia’s National Statement on Ethical Conduct in Human Research.

### Participants.

A convenience sample of 543 undergraduate students from the University of Western Australia (391 females) participated in exchange for partial course credit. Participants ranged in age from 19 to 56 y (mean = 24 y, SD = 6.84 y).

### Task and Procedure.

The task for each participant was to construct a paper plane that flew as far as possible using a single piece of A4 paper (1, 15, 19). Participants were randomly allocated to an eight-generation transmission chain. This design is widely used to experimentally study social learning (47–50).

Participants were randomly assigned to a condition: Individual Learning, 1-Model, 2-Model, or 4-Model. The Individual Learning condition (*n* = 31 participants, *n* = 31 chains, with the same subject participating eight times, once at each generation) was an asocial-learning condition. In the 1-Model (*n* = 128 participants, *n* = 16 chains, with one subject participating once at each generation), 2-Model (*n* = 128 participants, *n* = 8 chains, with two subjects participating once at each generation), and 4-Model (*n* = 256 participants, *n* = 8 chains, with four subjects participating once at each generation) conditions, each participant was randomly assigned to a position in the chain (1–8) and completed the task once, within a single generation, before being removed from the experiment. Testing took place in a large room that was 12 m long by 7.5 m wide. The room allowed us to test up to eight participants simultaneously (separated by visual barriers to eliminate the possibility of socially learning from sources other than those intended as part of the experimental design). To enable quick and accurate measurements of flight distance, we placed duct tape across the width of the testing room floor at 1-m intervals and marked on each the distance from the participant. Flight distance was measured using a tape measure, from the nearest taped marker to the paper plane.

Each participant received verbal and written instructions before commencing the experiment. Participants were seated at a table and given 5 min to construct a paper plane that would fly as far as possible using the A4 sheet of paper provided. Plane construction was restricted to folding the paper; ripping the paper or rolling it into a ball was not permitted. A stopwatch was placed on the table so that participants could keep track of the remaining time. In addition, the experimenter verbally informed participants when there was 1 min remaining, when there were 30 s remaining, and when their time was up. Next, participants flew their paper plane three times from a seated position in a high back chair (all chairs were set at an equal height). This was done to reduce the influence of differences in participant height, and to eliminate any “run-throw” or related techniques, restricting performance, as much as was possible, to the characteristics of the paper plane. For each paper plane, all three flight distances were recorded by the experimenter, and the longest flight distance was recorded alongside the plane on a separate piece of paper. The longest flight distance was representative of the paper plane, correlating strongly with the mean of the other two flight distances [r(758)=0.89,P<0.001]. The experimenter photographed each paper plane, before the plane and its associated maximum flight distance were made available to the next person(s) in the transmission chain.

At Generation 1, all participants constructed a paper plane without any opportunity for social learning. Hence, the conditions were identical at Generation 1. The conditions differed from Generation 2 onwards. In the Individual Learning condition, the same participants completed the paper plane task at each experimental generation. At Generation 2, participants in the Individual Learning condition were given 1.5 min to study the paper plane they had previously built and its associated maximum flight distance, after which both were removed. Participants were then given 5 min to build a plane and then flew the plane three times. The flight distances were recorded, and the maximum flight distance was placed alongside the plane on a separate piece of paper. This procedure was followed eight times, equivalent to the eight generations in the other conditions. A similar procedure was followed in the 1-Model condition. However, in this condition, each participant left the experiment after a single generation testing session and was replaced by a new participant who represented the next generation. The Generation 2 participant studied the paper plane produced by the Generation 1 participant and its associated maximum flight distance (for 1.5 min). The Generation 2 participant then produced his or her own paper plane (5 min) and flew it three times. The flight distances were recorded by the experimenter, and the plane and its maximum flight distance were made available to the Generation 3 participant and so on across the eight experimental generations.

In the 2-Model condition, Generation 2 participants viewed two planes, each constructed by a different Generation 1 participant, and their associated maximum flight distances. Each plane was separately viewed for 1.5 min (i.e., 3 min total viewing time) before being removed. Planes were randomly assigned to the participants and rotated every 1.5 min. The Generation 2 participants then constructed their own paper plane (5 min) and flew it three times. Their planes and their associated maximum flight distances were then passed to the Generation 3 participants. The same procedure was followed by participants in the 4-Model condition, but now with four planes and their associated maximum flight distances. Participants in this condition were given 1.5 min to separately view each plane (i.e., 6 min in total) before constructing a plane (5 min) and flying it three times. The planes and their maximum flight distances were then passed to the Generation 3 participants. This procedure continued across the eight experimental generations.

## Supplementary Material

Supplementary File
