# Supplementary material for: Increasing population size can inhibit cumulative cultural evolution
Source: Proc Natl Acad Sci U S A. 2019 Mar 14;116(14):6726–31. doi: 10.1073/pnas.1811413116 (PMC6452720; doi:10.1073/pnas.1811413116)
Supplement: Supplementary File [file pnas.1811413116.sapp.pdf]

Supporting Information: Paper Plane Complexity

Plane complexity was operationalized as the number of folds made to construct each paper plane. The plane complexity data was analyzed using a generalized linear mixed effects model, including by-chain random intercepts. Because the data are counts, a Poisson probability distribution was specified. The fixed effects (Condition, Generation) were centered prior to analysis.

The best fitting model specified Condition as a fixed effect without interaction (see **Table S1**, **Figure S1**). Plane complexity increased as population size increased (from 7.98, 8.68, 8.79 and 9.16 folds in the Individual Learning, 1-, 2- and 4-Model conditions respectively). There was no evidence of a statistical change in plane complexity over the experimental generations.

Table S1. Change in paper plane complexity over generations: Results from the generalized linear mixed effects model.

Fixed Effects	Estimate	Standard Error	z value	Pr(>|z|)
**Initial linear mixed effects model (BIC = 3454.1)**				
Condition	0.031	0.007	4.34	<.001
Generation	0.004	0.005	0.78	.433
Condition*Generation	- 0.003	0.003	-1.05	.292
**Final linear mixed effects model (BIC = 3442.5)**				
Condition	0.032	0.007	4.33	<.001

www.pnas.org/cgi/doi/10.1073/pnas.1811413116

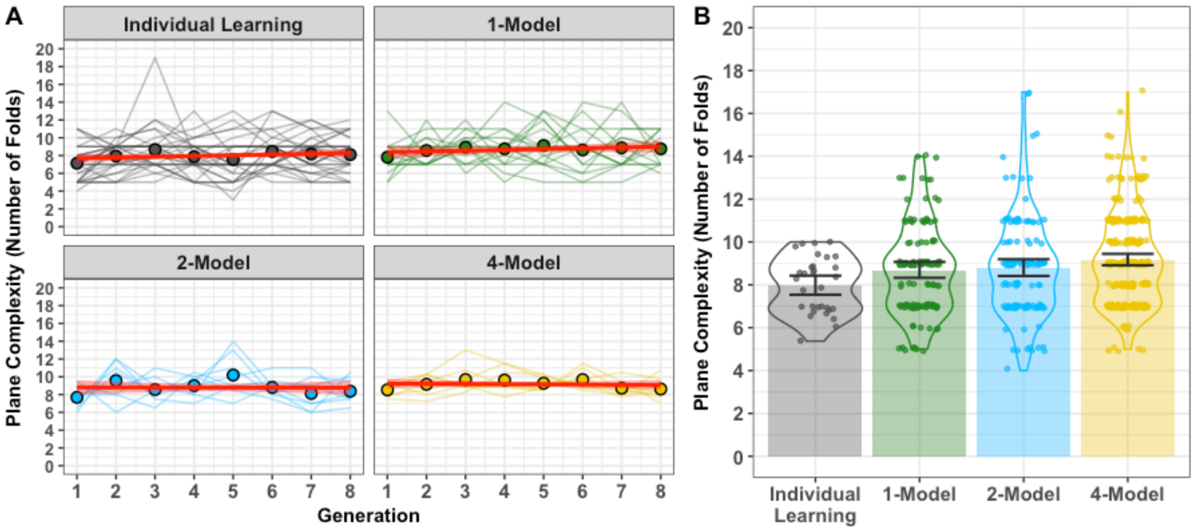

Figure S1. Panel A shows the change in paper plane complexity across the experimental generations in the different conditions (plotted for each chain). The dot points reflect the overall mean at each generation. The red straight line is the linear model fit and the light red shaded area is the bootstrapped 95% confidence interval. Panel B shows the plane complexity scores for each condition. The coloured bars indicate the overall mean for each condition and the dot points indicate the mean for each participant. Error bars are the bootstrapped 95% confidence intervals.
